# Effectiveness and mechanism of a 4-week online self-help mindfulness intervention among individuals with emotional distress during COVID-19 in China

**DOI:** 10.1186/s40359-022-00831-7

**Published:** 2022-06-13

**Authors:** Ruilin Ju, Wingsze Chiu, Yinyin Zang, Stefan G. Hofmann, Xinghua Liu

**Affiliations:** 1 Beijing Key Laboratory of Behavior and Mental Health, School of Psychological and Cognitive Sciences, Peking University, No. 5 Yiheyuan Road Haidian District, Beijing, People’s Republic of China; 2 Department of Clinical Psychology, Philipps-University Marburg, Marburg, Germany; 3 Department of Psychological and Brain Sciences, Boston University, 900 Commonwealth Avenue, 2nd Floor, Boston, MA 02215 USA

**Keywords:** Mindfulness, Online intervention, Self-help intervention, Emotional distress, COVID-19

## Abstract

**Background:**

Many people suffered from emotional distress especially during the COVID-19 pandemic. In order to alleviate emotional distress, more accessible psychological intervention programs, such as online intervention programs, are needed. The study aimed to investigate the efficacy and the potential mechanism of a 4-week, online, self-help mindfulness-based intervention to manage emotional distress during the COVID-19 pandemic between February 3 and May 20, 2020.

**Methods:**

A total of 302 individuals with high emotional distress completed a self-help mindfulness course, which lasted 30–60 min per day for 28 consecutive days. Participants who registered in the program later were included in the analyses as the control group (n = 315). Levels of mindfulness, perceived stress, emotional distress, anxiety and depression were assessed at baseline(T1), week 1(T2), week 2(T3), week 3(T4) and week 4(T5).

**Results:**

Significant Group by Time interaction effects were found on mindfulness, perceived stress, emotional distress, anxiety and depression (*p* < 0.001). Compared to the control group, the intervention group had a greater increase in changes of all outcome variables (*p* < 0.001). Random intercept cross-lagged analyses showed that compared with control group, mindfulness at T2 and T4 negatively predicted stress at T3 and T5, and mindfulness at T2 and T4 negatively predicted depression at T3 and T5 while depression at T3 predicted mindfulness at T4 in the mindfulness group.

**Conclusions:**

The results suggest that a 4-week self-help online mindfulness intervention improved mindfulness and reduced stress, emotional distress, anxiety and depression symptoms. Compared to the control group, changes in mindfulness preceded changes in stress, and mindfulness and depression reciprocally influenced each other during the intervention.

*Trial registration* Chinese Clinical Trial Registry: ChiCTR2000034539. Registered 9 July 2020—Retrospectively registered, http://www.chictr.org.cn/edit.aspx?pid=55721&htm=4.

**Supplementary Information:**

The online version contains supplementary material available at 10.1186/s40359-022-00831-7.

## Background

Depressive and anxiety disorders are common mental illnesses. The lifetime prevalence of depressive disorders and anxiety disorders in China are 6.8% and 7.6%, respectively [1]. In addition, many people suffered from emotional distress without meeting diagnostic criteria of depressive and anxiety disorders, especially during the COVID-19 pandemic [2]. In order to alleviate emotional distress, more accessible psychological intervention programs, such as online intervention programs, are needed. In the late 1970s, Kabat-Zinn introduced Mindfulness-based Stress Reduction (MBSR) into psychotherapy to help patients cope with stress, relieve pain, improve mood, and improve life comfort [3]. Some studies provided support for MBSR as a way to relieve psychological distress in the general population [4], such as negative emotions [5], anxiety [6] and recurrent depression [7, 8]. However, MBSR is usually carried out face to face, limiting its reach. Online self-help MBI is a promising approach, with a more accessible and convenient form to help more people with emotional distress, in particular during the COVID-19 pandemic. A survey conducted in the early days of the outbreak in China found that more than half of respondents rated the psychological impact as moderate-to-severe, and about one-third reported moderate-to-severe anxiety [9]. With vulnerable groups increasing during the COVID-19 epidemic, more accessible psychological interventions are needed. A number of studies and meta-analyses have examined the effectiveness of online mindfulness intervention [10–14]. For example, Spijkerman et al. [15] found that self-help mindfulness interventions had small but significant effects on mindfulness and psychological distress, including stress, anxiety and depression. In addition, a randomized controlled study found that self-help mindfulness-based intervention showed significant improvement in emotion regulation after intervention [16]. These mental health benefits suggest that online mindfulness interventions could be of assistance to more people.

So far, most of the online mindfulness interventions were conducted with MBSR, Mindfulness-Based Cognitive Therapy (MBCT) or Acceptance and Commitment Therapy (ACT) [15]. Here, we employed the Unified Protocol for the Treatment of Emotional Disorders (UP), which is a general manual for treating various emotional disorders to relieve emotional distress by emphasizing the adaptive and functional nature of emotions, facilitating greater tolerance of emotions, and identifying and correcting maladaptive attempts to regulate emotional experiences [17, 18]. Two recent meta-analyses suggested that this intervention method was an effective emotion regulation strategy associated with significant effects across various measures of depression and anxiety [19, 20]. Here, we aim to develop an online MBI targeting emotional distress by integrating theoretical understanding of emotional disorders and some practical approaches of UP and to examine its efficacy.

In addition to studying the efficacy of mindfulness-based interventions, it is also necessary to understand why a successful intervention works and how it can be optimized. Therefore, the current study will explore the underlying mechanism of the intervention [21]. So far, there have been three systematic reviews on the mechanism of mindfulness intervention [22–24]. The results showed that mindfulness was a significant mediator of the intervention. Mindfulness was defined as the capacity to be aware, to pay attention to the present moment, and to be accepting and nonjudgmental [25, 26]. Maintaining mindfulness means not to suppress or avoid emotional feelings, but to face and accept emotional feelings. Emotional distress can be reduced by gradually increasing the willingness and acceptance to experience strong emotional feelings, reducing the behaviors of avoiding and controlling emotional feelings, and improving cognitive flexibility. Numerous studies and reviews on offline mindfulness courses provide evidence suggesting that improvement in mindfulness is an important mediator of changes in psychological health outcomes [27–30]. In contrast, Labelle et al. [31] found that changes in mindfulness did not mediate the effects of MBSR on rumination and worry. For a variable to serve as a treatment mediator, there should be temporal precedence of the mediator in relation to the outcome [32]. This necessitates multiple assessments points. However, there were only three studies using multiple time points in exploring the mechanism role of mindfulness so far [33]. Therefore, research using multiple time points is needed to examine whether mindfulness is a mechanism of self-help mindfulness intervention programs.

In this study, we examined the efficacy and mechanism of a brand-new 4-week self-help online mindfulness intervention for emotional distress in Chinese adults. We hypothesized that a 4-week, self-help mindfulness intervention program would significantly improve mindfulness, stress, anxiety, depression and emotional distress during an early stage of the COVID-19 pandemic. We further predicted that in the mindfulness group, an increase in weekly mindfulness would predict the subsequent reduction in weekly anxiety, depression, emotional distress and stress as compared to the control group.

## Methods

### Procedures

We conducted three recruitment waves. The first and second waves began on February 3rd 2020 and February 18th 2020 respectively, and participants from these waves constituted the intervention group. The third-wave recruitment was held on April 14th 2020, and participants from the third wave served as a wait-list group.

The study was advertised on the website of the authors’ lab. Participants who met the criteria provided online informed consent before the baseline assessment and were invited into a WeChat group for receiving the assessment links and resolving any technical difficulties. Participants did not receive any compensation, and they were not asked to pay for taking part in this research.

### Participants

A total of 693 people completed an online registration questionnaire, which gathered demographic information, along with the Chinese version of the Kessler Psychological Distress Scale (K10) [34]. The self-help mindfulness intervention was provided as a psychological assistance for individuals with emotional distress after the outbreak of COVID-19 pandemic. Therefore, subjects could not be randomized to a control group at that time. For ethical reasons, the control group was formed in April instead.

Participants had to be between the ages of 18 to 65 and have a current Kessler Psychological Distress Scale score over 22 [35] to be included in the study. According to Andrews & Slade [36], scores on K10 are subsequently categorized into four levels: low (scores of 10–15); moderate (scores of 16–21); 'high' (scores of 22–29) and 'very high' (scores of 30–50). We set the K10 cut-off score to 22 because in this study we focused on the individuals with high and very high emotional distress. Participants were excluded from the study if they were diagnosed with a severe physical or mental illness (e.g., asthma, heart disease, schizophrenia, post-traumatic stress disorder, and bipolar disorder), or they had a history of alcohol or drug abuse, or severe trauma. This information was obtained from self-report registration questionnaire.

A total of 349 applications were excluded based on the study criteria, and 42 withdrew before the baseline assessment. As a result, 302 participants enrolled in the mindfulness course during this first and second enrollment wave.

A third wave of enrollment using the same recruitment procedure as above was conducted two months later, resulting in 452 new applicants. The exclusion criteria were the same as for the previous waves. After screening, the wait-list group consisted of a total of 315 participants. This group was offered to participate in the online self-help mindfulness course after the post-test had been completed.

**Fig. 1:**
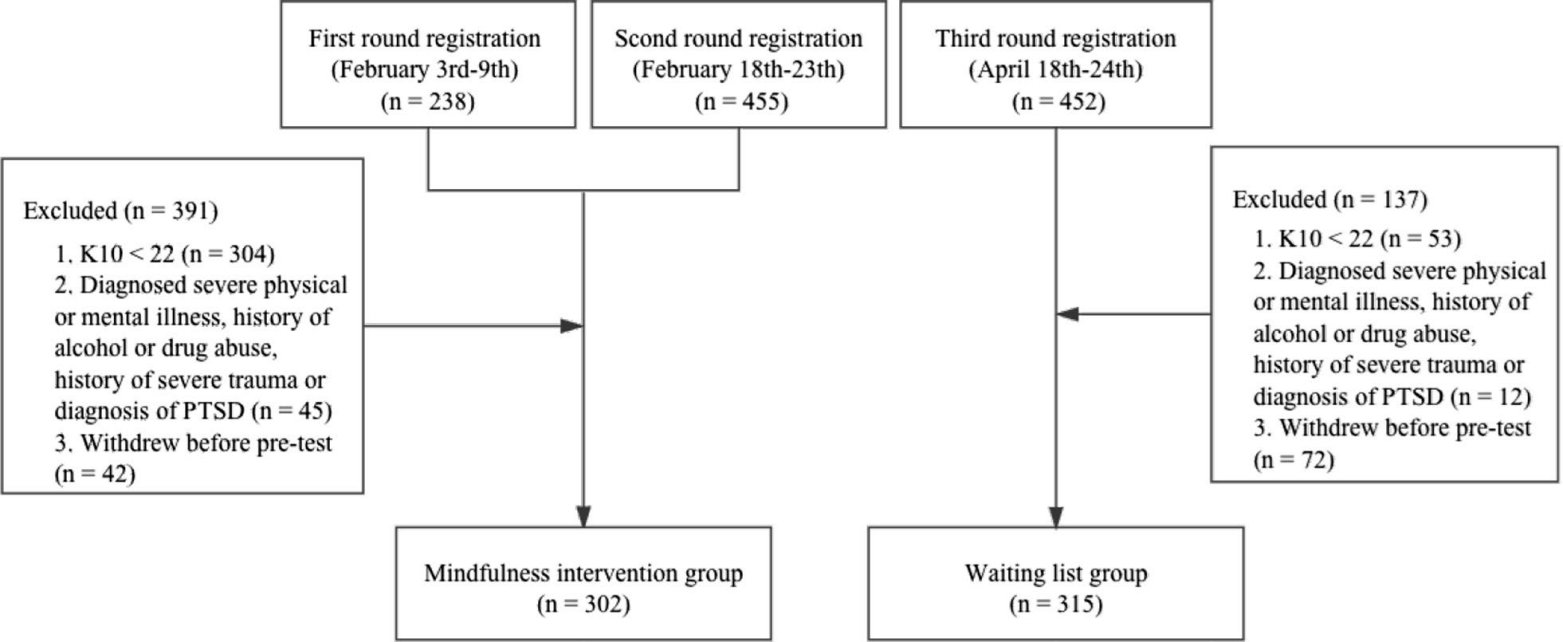
Participant flowchart

In all, 617 individuals participated in the present study (Fig. 1). Ethical approval for the study was obtained from Ethics Committee of School of Psychology and Cognitive Science of Peking University.

The intervention group was given access to the course immediately after one-week screening stage, while the control group was informed that they would be invited to join the course after a four-week waiting period. Participants completed assessments using an online survey platform at baseline(T1), week 1(T2), week 2(T3), week 3(T4) and week 4 (post-treatment, after all intervention sessions ended, T5) to measure mindfulness, perceived stress, anxiety, depression and emotional distress.

### The online mindfulness-based intervention

The “Mindfulness Intervention for Emotion Distress” self-help course was developed on the basis of MBSR [37] and UP [17]. The UP is an emotion-focused, cognitive behavioral intervention targeting the temperamental characteristics, particularly neuroticism and resulting emotion dysregulation, underlying all anxiety, depressive, and related disorders [38].

The course was delivered by a WeChat Mini Program, and it lasted 28 days with different content every day. Specifically, daily lessons consisted of three sections, including audio-guided mindfulness practice (15–45 min per practice), psychoeducation (basic knowledge of mindfulness and emotional distress, and frequently asked questions about mindfulness practice), and homework, in total requiring 30–60 min to finish. All formal (e.g., body scanning, mindful breathing, and mindful stretching) and informal (e.g., mindful tooth brushing) mindfulness practices were included in this course. In addition, some strategies of UP for transdiagnostic treatment of emotional disorders were introduced into this course by encouraging participants to stay with unpleasant experiences through exercises such as mindful stretching and quick breathing, identifying avoidance and emotion-driven behaviors, and completing challenging tasks. The course was designed and audio files were recorded by one of the authors, an experienced clinical psychologist with training and expertise in delivering mindfulness-based intervention and the UP. For more detailed information about the course, see Table 1.

**Table 1:** Overview of the online mindfulness-based intervention

	Mindfulness practices	Psychoeducation (audio and reading materials)	Other tasks
Week 1	Body scanning, mindful breathing	(1) Purpose and requirements of the course	Unpleasant events diary (starting from the fourth day)
		(2)The practice tips for mindfulness practices	
		(3) Three factors of emotion: thoughts, feelings, and behaviors	
		(4) Frequently asked questions about mindfulness practice	
Week 2	Mindful breathing, three-step breathing space, mindful stretching	(1) How emotions bring us distress? (from UP)	Unpleasant events diary
		(2) How to treat thoughts and unpleasant feelings? (from UP)	
		(3) Three-step breathing space	
		(4) Frequently asked questions about mindfulness practice	
Week 3	Mindful seating, Mindful walking	(1) How mindfulness works for emotional distress? (from UP)	Rapid breathing practice (from UP)
		(2) Recognizing two thought-traps (from UP)	
		(3) Recognizing emotion-driven actions and avoidance actions (from UP)	
		(4) What is rapid Breathing Practice? (from UP)	
		(5) Frequently asked questions about mindfulness practice	
Week 4	Mindful seating, body scanning	(1) What is challenging task and why it is important? (from UP)	Challenging task (facing one situation stimulating emotional distress) (from UP)
		(2) Common problems in mindfulness practice	
		(3) Review and summary	
		(4) Frequently asked questions about mindfulness practice	

Participants were invited to follow the daily content of this intervention program and record their practice and reflections under each lesson. Participants of each course were in a WeChat group and the course assistants were present for resolving any technical difficulties while using the program in WeChat on participants’ smart phone. Otherwise, the program was solely self-help. The participants could finish the course at any time and in their own home, which limited commuting time and gave the program high cost-effectiveness.

### Measures

#### Demographics

The following demographics information was collected at registration: sex, age, and whether participants were affected by the COVID-19 virus (select from the five possible responses:1 = I have the diagnosis or I am a suspected patient, 2 = my relatives or friends have the diagnosis or are suspected patients, 3 = diagnosis is present in my community or there are suspected patients in my community, 4 = my work is related to COVID-19 prevention; 5 = none of them; choosing 1, 2, 3, or 4, will be categorized as “COVID-19 related”).

#### Mindfulness skills

The Short Form of the Chinese Version of Five-factor Mindfulness Questionnaire (FFMQ-SF) [39] is a 20-item scale. It measures five facets of mindfulness skill, namely describing, observing, nonjudging to inner experience, nonreacting to inner experience and acting with awareness, on a 5-point Likert scale ranging from 5 (extremely comparable/similar) to 1 (not at all comparable/similar). In the current study, the FFMQ-SF showed good internal consistency, Cronbach’s alpha = 0.814.

#### Perceived stress

The Chinese Perceived Stress Scale (CPSS) [40] measures the stress participants experience in their lives. The 14-item scale asks participants to rate how often they have felt, or thought, that they had been out of control, overloaded and unpredictable during the last two weeks on a 5-point Likert-type scale from 0 (never) to 4 (very often). In the current study, the CPSS showed good internal consistency, Cronbach’s alpha = 0.886.

#### Emotional distress

The Chinese version of the 10-item Kessler Psychological Distress Scale (K10) [34] measures the frequency of anxiety and depression symptoms experienced in the past four weeks on a 5-point Likert-type scale ranging from 5 (all the time) to 1 (never). Scores on the K10 are subsequently categorized into four levels: low (scores of 10–15); moderate (scores of 16–21); 'high' (scores of 22–29) and 'very high' (scores of 30–50). We set the K10 cut-off score to 22 [36]. In current study, the K10 showed good internal consistency, Cronbach’s alpha = 0.924.

#### Anxiety

The Overall Anxiety Severity and Impairment Scale (OASIS) [41, 42] is a 5-item self-report scale that evaluates the frequency and severity of anxiety symptoms, the functional impairment related to these symptoms (i.e. school, work, home, or social impairment), and behavioral avoidance. Each item instructs respondents to select one of five responses that best describes their experiences over the past week. Response items are coded on a 5-point scale (0–4). The sum of the scores is used to obtain the total score, which can be a maximum of 20. In the current study, the OASIS showed good internal consistency, Cronbach’s alpha = 0.918.

#### Depression

The Overall Depression Severity and Impairment Scale (ODSIS) [41, 43] is a brief, 5-item, self-report scale to assess the severity and functional impairment associated with depressive symptoms. Items are coded on a 5-point scale (0–4). The sum of the scores is used to obtain the total score, which can be a maximum of 20. In the current study, the ODSIS showed good internal consistency, Cronbach’s alpha = 0.958.

### Preliminary data screening and data analysis

Missing data occurred within each assessment time point because some participants did not complete all waves of the survey. Missing data of each survey at Times 1, 2, 3, 4 and 5 were 2.32, 39.07, 51.66, 53.97 and 55.30% respectively for the intervention group, and they were 4.13, 9.52, 21.59, 18.10, and 13.33% respectively for the control group. We used Linear Mixed models to evaluate the effects, as this method allows all participants to be included in the analyses, regardless of missing data. When evaluating the mechanism, missing data were estimated using the full information maximum likelihood (FIML) estimation method in Mplus. Although several possible outliers were identified in each sample, exclusion of these participants did not appreciably alter the results. For this reason, none of the participants were excluded as outliers in either sample. Residual plots for each analysis revealed satisfactory adherence to the regression assumptions of normality, linearity, and homoscedasticity.

Data analysis was conducted using three statistical methods. First, we compared two waves and two groups respectively on baseline demographic and psychological outcomes using t-tests for continuous variables and χ2 tests for categorical variables. Second, repeated measurement analyses were conducted using the linear mixed model procedure of SPSS Statistics 26.0. We aimed to compare changes in the outcome variables between the intervention and control groups across the five time points. In each model, interactions of time and group as fixed effects were included. Paired sample t-tests were conducted in each group to examine whether there were significant score changes after the intervention or waiting period. Independent t-tests were conducted comparing changes of each outcome variable of the two groups. The effect sizes were calculated in both groups respectively using Cohen’s d statistics. The effect sizes were considered large when d = 0.8 or higher, medium when d = 0.5–0.8, and low when d = 0.2–0.5.

Third, we administered the random intercepts cross-lagged panel model (RI-CLPM) [44] using Mplus 8.0 [45] to examine the temporal relationship between mindfulness (measured by FFMQ-SF), and other psychological outcomes—perceived stress, emotional distress, anxiety, and depression (measured by CPSS, K10, OASIS and ODSIS). The RI-CLPM is an alternative to the widely used cross-lagged panel model (CLPM), which was criticized because it cannot disentangle within-person effects over time from between-person stable effects. By including a random intercept (i.e., a factor with all loadings constrained to 1), the RI-CLPM accounts for trait-like, time-invariant stability and thus partials out between-person variance and obtains the real within-person dynamics [44].

**Fig. 2:**
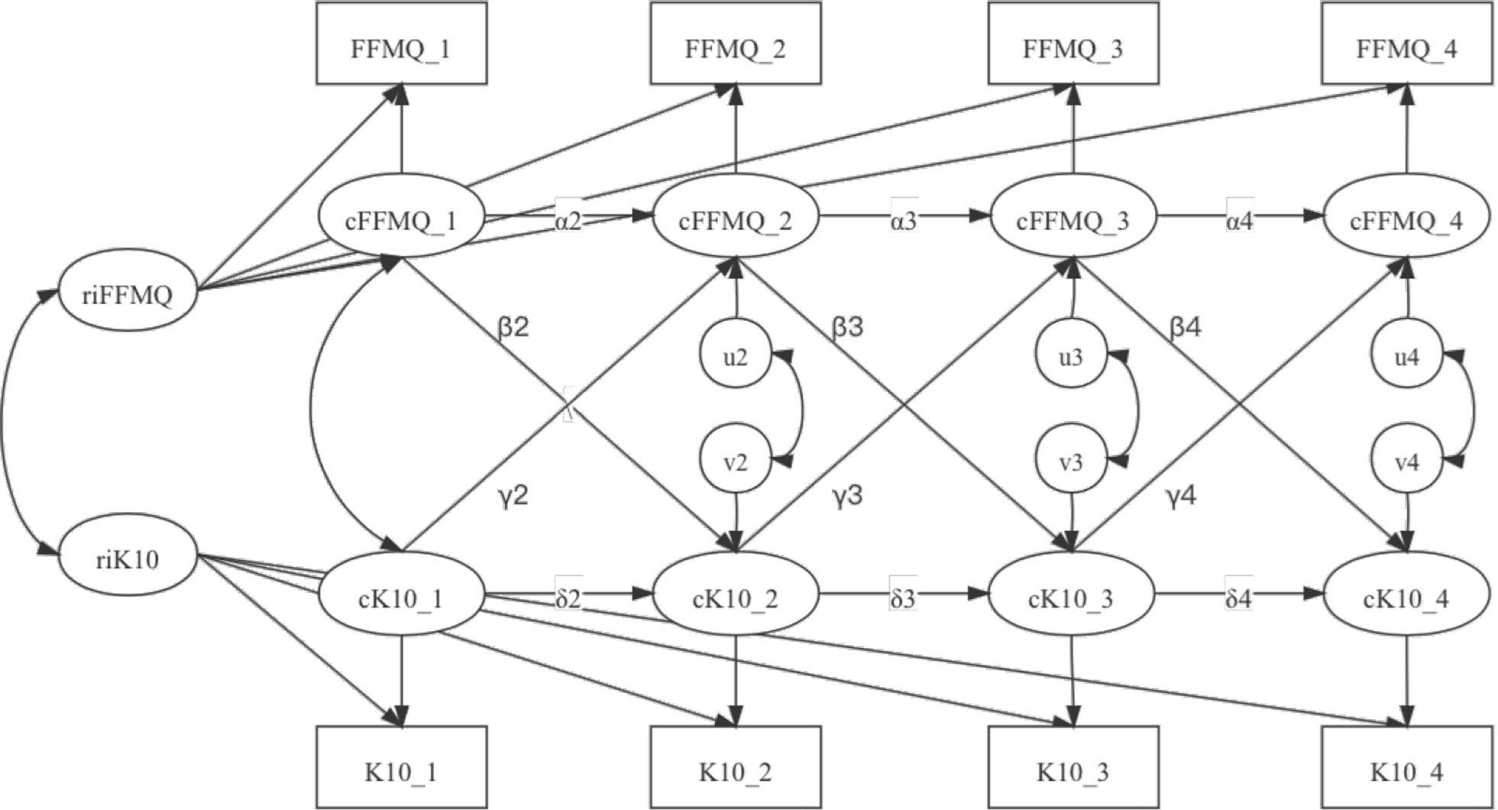
Random intercept cross-lagged panel model (RI-CLPM). *Note*: The RI-CLPMs estimates the potential reciprocal relationships between mindfulness (FFMQ) and four psychological outcomes, which are perceived stress (CPSS), emotional distress (K10), anxiety (OASIS), and depression (ODSIS) for the four waves of data. The RI-CLPM of FFMQ and K10 is taken as an example here. Each observed score is decomposed into two parts: a within-person part and a between-person part. The cFFMQ and cK10 factors represent the within-person part of the outcomes. The two random intercepts, riFFMQ and riK10 capture the between-person part. ri, random intercept; c, within-person centered variables

Figure 2 shows an illustration of the RI-CLPM examined in the current study. The random intercepts reflect an individual’s average, stable level of mindfulness and emotional distress. The autoregressive parameters α2–α5 and δ2–δ5 relate to the degree of within-person carry-over effects, thus showing whether deviations from one’s own expected emotional distress or mindfulness score at one measurement occasion carry over to the next occasion. The cross-lagged parameters β2–β5 and γ2–γ5 refer to relationships at the within-person level and can be interpreted as the extent to which changes in an individual’s deviations from the expected score of one variable (e.g., emotional distress) are predicted by deviations from the expected score of another variable (e.g., mindfulness) at the previous measurement occasion after adjusting for the carry-over effects.

To study group differences, we performed multiple group analyses. We compared a multiple group version of the RI-CLPM in which there are no constraints across the groups, with a model in which the lagged regression coefficients are constrained to be identical across the groups. If the chi-square difference test indicates that this constraint cannot be imposed, this implies that (some of) the lagged coefficients differ across the groups: The lagged effects of the variables on each other depend on the level of the grouping variable. In contrast, when the equality constraints on the lagged parameters across the groups hold, this implies there is no moderation effect [46]. Models that fit well are indicated by CFIs and TLI > 0.90, SRMR < 0.10 and RMSEAs < 0.08 [47].

## Results

### Descriptive statistics

Baseline demographic and psychological variables for each group are presented in Table 2. The sample was 78.2% female, and the mean age was 31.528 years (SD = 9.504). There were 33.22% of the sample affected by COVID-19. Samples in wave 1 and wave 2 were equivalent in age (*t*_300_ = 1.117, *p* = 0.379) and sex (*χ*2 = 0.990, *p* = 0.343). They did not show significant differences in the initial CPSS (*t*_292_ = 0.826, *p* = 0.711), FFMQ total score (*t*_292_ = 0.947, *p* = 0.973), or any FFMQ subscales (*t*_292_ = 0.265–0.391, *p* = 0.234–0.977), but significant group differences on K10 (*t*_292_ = 0.778, *p* = 0.039), OASIS (*t*_292_ = 0.424, *p* = 0.029), and ODSIS (*t*_292_ = 0.509, *p* = 0.011).

**Table 2:** Participant demographics and psychological characteristics in baseline

	Mindfulness (n = 302)					Wait list (n = 315)	*t/χ2`*	*p*
	Wave 1 (n = 120)	Wave 2 (n = 182)	t/χ2	*p*	Total	N(%)/Mean(SD)		
	N(%)/Mean(SD)	N(%)/Mean(SD)						
Sex			0.900	0.343			0.803	0.370
Male	21 (17.5)	40 (22)			61 (20.2)	73 (23.2)		
Female	99 (82.5)	142 (78)			241 (79.8)	242 (76.8)		
COVID-2019 related			3.713	0.054			66.403	0.000
Yes	67 (55.8)	81 (44.5)			148 (49)	57 (18.1)		
No	53 (44.2)	101 (55.5)			154 (51)	258 (81.9)		
Age	32.08 (9.27)	30.53 (8.52)	1.499	0.135	31.15 (8.84)	31.90 (10.10)	− 0.979	0.328
K10	28.80 (6.31)	26.81 (6.16)	2.688	0.008	27.62 (6.29)	27.60 (6.85)	0.030	0.976
CPSS	33.44 (6.91)	32.75 (7.15)	0.815	0.416	33.03 (7.05)	32.95 (6.86)	0.141	0.888
OASIS	9.60 (3.67)	8.46 (3.55)	2.653	0.008	8.92 (3.63)	8.68 (3.54)	0.838	0.403
ODSIS	7.08 (4.34)	5.55 (3.92)	3.158	0.002	6.17 (4.15)	6.18 (4.46)	− 0.034	0.973
FFMQtotal	57.65 (7.46)	57.87 (8.08)	0.407	0.812	57.78 (7.82)	58.19 (8.08)	− 0.628	0.530
FFMQobs	12.13 (3.07)	12.35 (3.14)	− 0.617	0.538	12.26 (3.11)	12.54 (3.45)	− 1.032	0.302
FFMQdes	11.73 (2.64)	11.46 (2.95)	0.798	0.426	11.57 (2.83)	11.72 (2.97)	− 0.619	0.536
FFMQact	11.45 (2.77)	11.53 (2.83)	− 0.233	0.816	11.50 (2.80)	11.34 (3.28)	0.636	0.525
FFMQnj	11.94 (2.71)	12.31 (2.58)	− 1.174	0.241	12.16 (2.63)	11.97 (2.71)	0.839	0.402
FFMQnrt	10.40 (2.52)	9.94 (1.65)	1.859	0.064	10.13 (2.06)	10.62 (2.39)	− 2.678	0.008

*CPSS* Chinese Perceived Stress Scale, *K10* Kessler Psychological Distress Scale, *OASIS* Overall Anxiety Severity And Impairment Scale, *ODSIS* Overall Depression Severity and Impairment Scale, *FFMQtotal* total score of Short Form of Chinese Version of Five-factor Mindfulness Questionnaire, *FFMQobs* observe subscale of Short Form of Chinese Version of Five-factor Mindfulness Questionnaire, *FFMQdes* describe subscale of Short Form of Chinese Version of Five-factor Mindfulness Questionnaire, *FFMQact* act with awareness subscale of Short Form of Chinese Version of Five-factor Mindfulness Questionnaire, *FFMQnj* nonjudge subscale of Short Form of Chinese Version of Five-factor Mindfulness Questionnaire, *FFMQnrt* nonreact subscale of Short Form of Chinese Version of Five-factor Mindfulness Questionnaire

There were no significant differences between groups on sex (*χ*2 = 0.803, *p* = 0.370), age (*t*_615_ = -0.979, *p* = 0.328), K10 (*t*594 = 0.030, *p* = 0.976), OASIS (*t*_594_ = 0.838, *p* = 0.403), ODSIS (*t*_594_ =  − 0.034, *p* = 0.973), FFMQ total score (*t*_594_ =  − 0.682, *p* = 0.530), and four of the subscales of FFMQ (i.e., *observation, describing, act with awareness,* and *non-judgement*, see Table 1 for details), but significant differences on *non-reactivity* (*t*_594_ =  − 2.678, *p* = 0.008). The descriptive statistics for the dependent variables of all time points are shown in Table 2.

There were 61 (20.20%) participants who completed all sessions, and 146 (48.34%) participants completed at least 17 out of the 28 sessions, which is the criteria for course completion. A review of self-help mindfulness intervention also showed 48% of participants could meet study defined intervention engagement or completion criteria [48].

### Linear mixed model

Results of linear mixed effects model analyses comparing two groups are displayed in Table 3. Baseline scores of the FFMQ—*non-reactivity* facet was set as covariate because there was significant difference between the two groups. There were significant Group by Time interaction effects for scores of K10 (*t*_1207.76_ = 11.67, Cohen’s *d* =  − 0.906, *p* < 0.001), FFMQ (*t*_1212.18_ =  − 14.16, Cohen’s *d* = 0.895, *p* < 0.001), CPSS (*t*_1180.20_ = 11.35, Cohen’s *d* =  − 0.851, *p* < 0.001), OASIS (*t*_1117.67_ = 7.55, Cohen’s *d* =  − 0.581, *p* < 0.001), ODSIS (*t*_1138.25_ = 6.99, Cohen’s *d* =  − 0.472, *p* < 0.001), *observing* (*t*_1144.75_ =  − 8.44, Cohen’s *d* = 0.543, *p* < 0.001), *describing* (*t*_1153.76_ =  − 5.54, Cohen’s *d* = 0.336, *p* < 0.001), *act with awareness* (*t*_1204.44_ =  − 9.38, Cohen’s *d* = 0.534, *p* < 0.001), *non-judgment* (*t*_1196.64_ =  − 5.58, Cohen’s *d* = 0.473, *p* < 0.001), *non-reactivity* (*t*_1165.22_ =  − 7.52, Cohen’s *d* = 0.724, *p* < 0.001).

**Table 3:** Results of linear mixed models comparing intervention group and control group

	Parameter	Estimate	Std. error	df	t	Sig	95% Confidence Interval	
							Lower bound	Upper bound
K10	Time	− 3.31	0.22	1231.25	− 15.33	< 0.001	− 3.73	− 2.89
	Group	− 0.65	0.61	988.59	− 1.08	0.281	− 1.84	0.54
	Time * Group	1.48	0.13	1207.76	11.67	< 0.001	1.23	1.73
CPSS	Time	− 3.35	0.22	1202.56	− 15.55	< 0.001	− 3.77	− 2.93
	Group	− 1.67	0.63	977.75	− 2.66	0.008	− 2.91	− 0.44
	Time * Group	1.43	0.13	1180.20	11.35	< 0.001	1.18	1.68
FFMQ	Time	4.27	0.24	1232.70	17.85	< 0.001	3.80	4.74
	Group	2.51	0.75	906.15	3.36	0.001	1.04	3.97
	Time * Group	− 1.98	0.14	1212.18	− 14.16	< 0.001	− 2.26	− 1.71
OASIS	Time	− 1.56	0.12	1142.39	− 12.75	< 0.001	− 1.80	− 1.32
	Group	− 0.68	0.33	995.25	− 2.08	0.038	− 1.33	− 0.04
	Time * Group	0.54	0.07	1117.67	7.55	< 0.001	0.40	0.68
ODSIS	Time	− 1.22	0.13	1162.26	− 9.64	< 0.001	− 1.47	− 0.97
	Group	− 0.39	0.37	1001.45	− 1.04	0.298	− 1.12	0.34
	Time * Group	0.52	0.07	1138.25	6.99	< 0.001	0.37	0.66
FFMQobs	Time	0.95	0.09	1166.47	10.08	< 0.001	0.76	1.13
	Group	0.93	0.29	947.12	3.22	0.001	0.36	1.49
	Time * Group	− 0.46	0.05	1144.75	− 8.44	< 0.001	− 0.57	− 0.36
FFMQdes	Time	0.64	0.09	1175.95	7.46	< 0.001	0.47	0.81
	Group	0.31	0.26	923.72	1.22	0.223	− 0.19	0.82
	Time * Group	− 0.28	0.05	1153.79	− 5.54	< 0.001	− 0.38	− 0.18
FFMQact	Time	1.07	0.09	1230.26	11.55	< 0.001	0.89	1.25
	Group	0.35	0.27	998.38	1.31	0.191	− 0.17	0.87
	Time * Group	− 0.51	0.05	1204.44	− 9.38	< 0.001	− 0.62	− 0.40
FFMQnj	Time	0.74	0.10	1223.97	7.26	< 0.001	0.54	0.94
	Group	0.07	0.26	1103.90	0.26	0.794	− 0.44	0.57
	Time * Group	− 0.33	0.06	1196.64	− 5.58	< 0.001	− 0.45	− 0.22
FFMQnrt	Time	0.82	0.08	1192.21	9.87	< 0.001	0.66	0.98
	Group	1.05	0.20	1049.38	5.14	< 0.001	0.65	1.44
	Time * Group	− 0.37	0.05	1165.22	− 7.52	< 0.001	− 0.46	− 0.27

Results of paired t-test analyses are displayed in Table 4. Within the mindfulness group, a significant reduction from baseline to post-intervention was found in the K10, CPSS, OASIS and ODSIS scores (*p* < 0.001), as well as a significant increase in the FFMQ and its subscales (*p* < 0.001). However, significant changes were also found in the control group in all outcome variables (*p* < 0.001) except two FFMQ subscales, namely *observing* and *act with awareness* (*ps* > 0.05). When comparing changes in the outcome variables between the intervention and the control group, there were significant differences (*p* < 0.05), with the intervention group showing greater improvement on each of the variables. Besides, significant differences were also found in the post-tests between the two groups.

**Table 4:** Baseline values, post values and paired-t tests of intervention group and control group

	Intervention group			Control group			Cohen's*d*
	Pre	Post	*p*	Pre	Post	*p*	
K10	27.62 (6.29)	20.19 (6.44)	< 0.001	27.6 (6.85)	26.14 (7.58)	< 0.001	− 0.91
FFMQ	57.78 (7.82)	66.43 (9.94)	< 0.001	58.19 (8.1)	59.7 (9.29)	< 0.001	0.90
CPSS	33.03 (7.05)	24.95 (7.88)	< 0.001	32.95 (6.86)	30.79 (7.56)	< 0.001	− 0.85
OASIS	8.92 (3.63)	4.78 (3.16)	< 0.001	8.68 (3.54)	6.62 (4.14)	< 0.001	− 0.58
ODSIS	6.17 (4.15)	3.18 (3.19)	< 0.001	6.18 (4.46)	5.23 (4.57)	< 0.001	− 0.47
FFMQobs	12.26 (3.11)	14.1 (3.52)	< 0.001	12.54 (3.45)	12.59 (3.47)	0.815	0.54
FFMQdes	11.57 (2.83)	12.93 (3.08)	< 0.001	11.72 (2.97)	12.1 (3.28)	0.017	0.34
FFMQact	11.5 (2.8)	13.41 (2.94)	< 0.001	11.34 (3.28)	11.62 (3.35)	0.151	0.53
FFMQnj	12.16 (2.63)	13.76 (3.16)	< 0.001	11.97 (2.78)	12.29 (3.01)	0.047	0.47
FFMQnrt	10.13 (2.06)	12.24 (2.72)	< 0.001	10.62 (2.39)	11.11 (2.64)	0.005	0.72

### Random intercepts cross-lagged panel model

Four RI-CLPMs each tested the potential causal and reciprocal relationships between FFMQ and K10, CPSS, OASIS and ODSIS. We performed multiple group analyses to investigate group differences in lagged regression coefficients. The results of model fit and model comparison was shown is Table 5. All model fits of the unconstrained and constrained model were good (CFI = 0.986–0.994; TFI = 0.978–0.991; SRMR = 0.062–0.082; RMSEA = 0.037–0.058). Two of the constrained models had a significantly worse model fit compared with the unconstrained models (FFMQ-CPSS: Δ*χ2* = 27.440, Δdf = 16, *p* < 0.05; FFMQ-ODSIS: Δ*χ2* = 30.019, Δdf = 16, *p* < 0.05), indicating that the lagged effects for individuals in the mindfulness group and control group were different. The chi-square difference tests of other two nested models were not significant (FFMQ-K10: Δχ2 = 12.062, Δdf = 16, *p* = 0.740; FFMQ-OASIS: Δ*χ2* = 18.728, Δdf = 16, *p* = 0.283), which implied that imposing the constraints is tenable: The lagged effects for individuals in different group appear to be the same in these two models.

**Table 5:** Model fit and model comparisons

Model	*χ2*	df	Δχ2	Δdf	*p*	CFI	TLI	SRMR	RMSEA(CI)
FFMQ-CPSS									
Unconstrained model	86.026	42				0.990	0.979	0.062	0.058 (0.041, 0.076)
Constrained model	113.466	58	27.440	16	0.037	0.988	0.981	0.079	0.056 (0.040, 0.071)
FFMQ-ODSIS									
Unconstrained model	82.749	42				0.990	0.978	0.074	0.056 (0.038, 0.074)
Constrained model	112.768	58	30.019	16	0.018	0.986	0.979	0.082	0.055 (0.040, 0.070)
FFMQ-K10									
Unconstrained model	70.243	42				0.993	0.985	0.065	0.047 (0.026, 0.065)
Constrained model	82.305	58	12.062	16	0.740	0.994	0.991	0.075	0.037 (0.015, 0.054)
FFMQ-OASIS									
Unconstrained model	80.488	42				0.990	0.979	0.069	0.055 (0.036, 0.072)
Constrained model	99.216	58	18.728	16	0.283	0.989	0.984	0.077	0.048 (0.031, 0.064)

*CFI* comparative fit index, *TLI* Tucker–Lewis index, *SRMR* the standardized root mean square residual, *RMSEA* root mean square error of approximation

Standardized path coefficients of four RI-CLPMs are presented in Table 6. In the mindfulness intervention group, the FFMQ at T2 and T4 predicted less CPSS at T3 and T5 respectively *(β* = -0.0359, *p* < 0.05; *β* = -0.0574, *p* < 0.001). The FFMQ at T2 and T4 negatively predicted ODSIS at T3 and T5 (*β* =  − 0.462, *p* < 0.001; *β* = -0.475, *p* < 0.01), whereas K10 negatively predicted the FFMQ between T3 and T4 (*β* =  − 0.415, *p* < 0.01). The FFMQ at T4 predicted less OASIS at T5 *(β* =  − 0.352, *p* < 0.01; *β* =  − 0.574, *p* < 0.001). Other lagged paths were not statistically significant. In the RI-CLPM of the FFMQ and K10, none of the cross-lagged paths were significant.

**Table 6:** Standardized path coefficients of RI-CLPM between FFMQ and other psychological variables

	Intervention group				Control group			
	W1–W2	W2–W3	W3–W4	W4–W5	W1–W2	W2–W3	W3–W4	W4–W5
*FFMQ-CPSS*								
Stability paths								
FFMQ	− 0.077	0.219	0.463**	0.682***	− 0.206	0.107	0.437***	0.617***
CPSS	0.088	− 0.034	0.046	0.221	0.131	0.313**	0.473***	0.374***
Cross-lagged paths								
FFMQ → CPSS	0.130	− 0.359*	− 0.320	− 0.574***	− 0.044	− 0.099	− 0.053	− 0.184*
CPSS → FFMQ	− 0.042	− 0.032	− 0.156	− 0.042	0.130	− 0.302*	− 0.203*	0.012
*FFMQ-ODSIS*								
Stability paths								
FFMQ	0.095	0.247	0.311	0.673***	− 0.031	0.275*	0.527***	0.578***
ODSIS	0.368***	0.211	0.235	0.017	0.120	0.111	0.184	0.242*
Cross-lagged paths								
FFMQ → ODSIS	− 0.011	− 0.462**	− 0.231	− 0.475**	− 0.312*	0.076	0.088	0.266**
ODSIS → FFMQ	− 0.128	− 0.112	− 0.415*	− 0.089	− 0.130	0.006	0.161	0.071
*FFMQ-K10*								
Stability paths								
FFMQ	− 0.194	0.153	0.552***	0.674***	− 0.183	0.195	0.497***	0.548***
K10	0.106	0.445***	0.492***	0.650***	0.118	0.121	0.353	0.452***
Cross-lagged paths								
FFMQ → K10	− 0.065	− 0.024	− 0.065	− 0.107	0.060	− 0.167	− 0.158	− 0.108
K10 → FFMQ	0.122	− 0.213	− 0.061	− 0.071	− 0.067	− 0.079	− 0.089	− 0.108
*FFMQ-OASIS*								
Stability paths								
FFMQ	− 0.036	0.184	0.375*	0.709***	− 0.124	0.208	0.524***	0.581***
OASIS	0.337**	0.152	0.351	0.266	0.210	0.345***	0.524***	0.502***
Cross-lagged paths								
FFMQ → OASIS	0.176	− 0.290	− 0.158	− 0.352**	0.054	− 0.108	0.047	0.091
OASIS → FFMQ	0.080	− 0.028	− 0.280	0.003	0.132	0.012	0.016	0.004

^***^p < .001; **p < .01; *p < .05

We further tested the reciprocal relationships between five subscales of FFMQ and K10, CPSS, OASIS and ODSIS. For the *observing* subscale, only the lagged effects between *observing* and ODSIS showed a significant group difference (Δ*χ2* = 26.956, Δdf = 16, *p* < 0.05), In the mindfulness intervention group, *observing* at T2 and T4 predicted less ODSIS at T3 and T5, respectively (*β* =  − 0.396, *p* < *0*.05; *β* =  − 0.300, *p* < 0.05), whereas ODSIS at T3 predicted less *observing* at T4 (*β* =  − 0.567, *p* < 0.01). For the *describing* subscale, only the lagged effects between *describing* and CPSS showed a significant difference (Δ*χ2* = 37.005, Δdf = 16, *p* < 0.01). *Describing* at T3 and T4 predicted less CPSS at T4 and T5 respectively (*β* =  − 0.679, *p* < 0.001; *β* =  − 0.411, *p* < 0.001) in the intervention group. For the *act with awareness* subscale, the lagged effects of two groups showed significant difference in the relationship between *act with awareness* and CPSS (Δ*χ2* = 40.920, Δdf = 16, *p* < 0.01). In the mindfulness intervention group, *act with awareness* at T4 predicted less CPSS at T5 (*β* =  − 0.321, *p* < 0.001). For *non-judgment subscale*, *non-judgment,* and CPSS and K10, the lagged effects of two groups show significant difference in the relationship between non-judgment and CPSS, as well as *non-judgment* and K10 (Δ*χ2* = 27.764, Δdf = 16, *p* < 0.05; Δ*χ2* = 33.362, Δdf = 16, *p* < 0.01). None of the lagged paths was significant between *non-judgment* and CPSS. *Non-judgment* at T2 predicted less K10 at T3 (*β* =  − 0.233, *p* < 0.05), while K10 at T3 predicted less *non-judgment* at T4 (*β* =  − 0.220, *p* < 0.05). For the *non-reactivity* subscale, the lagged effects of three models showed significant group differences (*non-reactivity*-CPSS: Δ*χ2* = 45.276, Δdf = 16, *p* < 0.001; *non-reactivity*-K10: Δ*χ2* = 32.496, Δdf = 16, *p* < 0.01; *non-reactivity*-ODSIS: Δ*χ2* = 29.893, Δdf = 16, *p* < 0.05). In the mindfulness intervention group, *non-reactivity* at T3 and T4 predicted less CPSS at T4 and T5 respectively (*β* =  − 0.271, *p* < 0.05; *β* = -0.291, *p* < 0.01), whereas CPSS at T4 predicted less *non-reactivity* at T5 (*β* =  − 0.497, *p* < 0.001). K10 at T4 negatively predicted *non-reactivity* at T5 (*β* = -0.401, *p* < 0.001). ODSIS at T4 negatively predicted *non-reactivity* at T5 (*β* =  − 0.305, *p* < 0.01). For more detail on the model fits and standardized path coefficients, see Additional file 1: Tables s1–s5.

## Discussion

This study examined the efficacy of an online self-help MBI course in alleviating psychological symptoms such as perceived stress, emotional distress, anxiety, and depression symptoms in a sample of Chinese individuals with emotional distress during the early stage of the COVID-19 pandemic. Our results showed that the intervention group demonstrated a significant improvement in mindfulness and reductions in stress, anxiety and depression symptoms compared to the control group, supporting the application of the 4-week, online, self-help mindfulness intervention for individuals with emotional distress. Furthermore, we found that compared to the control group, changes in mindfulness preceded changes in stress, and that mindfulness and depression might reciprocally influence each other during the intervention.

With respect to the effects of the self-help mindfulness-based intervention on emotional distress and mental health, the present study found a significant Group by Time interaction effect for mindfulness, emotional distress, stress, anxiety and depression, with significant improvements in the changes of all outcome variables in the intervention group compared to waiting-list group, suggesting the effect of the online mindfulness-based intervention on mitigating anxiety, stress, depression and enhancing mindfulness. Other RCTs have shown similar results. For example, Henriksson et al. [49] highlighted that an 8-week, web-based mindfulness program had a significant Group by Time interaction on reduced affective distress and increased mindfulness on individuals with chronic pain. Jung et al. [50] found that after finishing an online MBT program for 8–10 min a day for 8 weeks, significant time by group interaction effects were found with respect to stress and negative affect. The results of the current study add to prior findings on the effectiveness of self-help mindfulness program, indicating that self-help mindfulness is a promising psychological intervention for mental health.

Surprisingly, we found that emotional distress, anxiety, depression and stress decreased significantly in the control group during their 4-week waiting period. One possible explanation is the positive effects from the anticipation of treatment, which might have alleviated the emotional distress to some extent. Another explanation is the turnaround of the pandemic in China. With strict prevention and medical control, the pandemic in China gradually stabilized during April 2020, during the waiting period of the control group. Reduction in health risk factors in the environment may have reduced negative emotions in control group participants, resulting in fewer health concerns and psychological problems.

Regarding the relationship between mindfulness and stress, mindfulness at T2 and T4 negatively predicted stress at T3 and T5 in the mindfulness group, as compared to the control group. This is consistent with the finding by Baer et al. [51], which suggested that the increase of mindfulness in week 3 significantly predicted the decrease of stress in week 8. The pattern of findings generally aligns with the widely held view that mindfulness training should increase the ability to respond mindfully to the experiences, which should lead to improvement in perceived stress.

We also found the mindfulness at T2 and T4 negatively predicted depression at T3 and T5, whereas depression at T3 negatively predicted mindfulness at T4 during the intervention. Our results showed that these findings are partially consistent with those obtained in previous studies where mindfulness predicted less depression [52, 53]. However, our results may also suggest that the decrease of depression could in turn predict an increase of mindfulness from T3 to T4. In other words, it is possible that an increase in mindfulness and a decrease in depression is due to a strong positive feedback loop between these two variables during the intervention.

Regarding the associations between mindfulness facets and emotional distress, significant group differences were found only in the *non-judgment* and *non-reactivity* subscales. *Non-judgment* predicted a decrease of emotional distress from T2 to T3, whereas emotional distress reversely predicted *non-judgment* from T3 to T4. The decrease of emotional distress predicted the increase of *non-reactivity* from T4 to T5. The heightened acceptance attitude for the *present* experience may alleviate emotional distress, which may then facilitate one’s ability to respond flexibly to negative thoughts and feelings without judgment.

No significant group differences were observed in the associations between mindfulness facets and anxiety. The significant improvements of mindfulness and anxiety in the control group may account for the non-significant group difference. Nevertheless, mindfulness was found to predict anxiety from T4 to T5 during the intervention. A similar pattern was also observed in the relationship between other subscales (i.e. observing, describing, and act with awareness) and anxiety. The unidirectional relationship between mindfulness and anxiety is consistent with the results of several studies suggesting that improvements in mindfulness predicted improvements in anxiety in non-clinical samples [54, 55].

Low adherence in the current study needs attention. A review of randomized controlled studies based on phone interventions for mental health suggests that attrition rate was 24.1% at short-term follow up and 35.5% at longer-term follow up [56]. In the current study, only 48.34% of the participants completed at least 17 out of the 28 sessions. There are many potential causes. One explanation is that less mentoring may lead to lower compliance, especially when the course does not have a professional teacher who can meet the students face to face to motivate them. Besides, unlike the traditional eight-week practice, our intervention required a daily commitment of time, which increased the difficulty of completion and affected adherence. The results need to be interpreted with caution due to the high attrition rate.

### Limitations

Several limitations of the current study need to be acknowledged. First, the current study did not have a randomized control group. The control and intervention groups in this study were not recruited at the same time and randomly assigned, which limits the interpretation of the results. Second, a waiting control group was used rather than an active control group, making it difficult for us to draw conclusions about the role of the UP. Future research should use active control groups to remedy this problem and examine the extent to which the UP contributes to the effectiveness of the intervention. Third, whether participants met the inclusion criteria was only based on subjective report, and no evaluator assessment instruments were employed. The reason for this was because we conducted the course for the purpose of psychological assistance and hoped to simplify the process so that more people could benefit. Fourth, the strict exclusion criteria made the inclusion rate of the intervention group less than 50%, which reduced the degree of interpretation for conclusions in the population. Fifth, the attrition rate of the current study is high, with only 48.34% of the participants completing at least 17 out of the 28 sessions. A previous study had discussed seven factors that are important in establishing and maintaining a regular mindfulness practice during an 8-week mindfulness course [57]. Future research can explore what factors might contribute to the adherence of self-help courses. In addition, the lack of follow-up limits the interpretation of the intervention's long-term efficacy A further limitation was the over-reliance on scale measurements. All variables were collected through self-report scales, and the practice effect caused by repeating the measures could have produced some bias in the results.

In spite of these limitations, our study demonstrated that an online self-help mindfulness intervention can reduce negative affect and perceived stress in individuals with emotional distress during the COVID-19 pandemic. Requiring few therapist resources and publicly accessible, this brief course is a promising intervention for individuals in emotional distress. More importantly, the current study was one of few that employed multiple time point assessments and cross-lagged analysis to explore the causal relationship between process variable (mindfulness) and psychological outcomes in an online self-help MBI, thereby extending the findings of existing studies that used multiple time points assessments to test the relationship between mindfulness and other mental health outcomes [13, 50, 58].

For future research, follow up data should be collected to explore whether changes in mindfulness and other mental health outcomes are maintained over a longer period. Comparing an online self-help mindfulness group with a randomized control group, or even an active control group (e.g., progressive muscle relaxation or stress management program), should also be addressed.

## Conclusions

To conclude, the finding from the current study suggest that an online self-help mindfulness intervention course is effective for improving perceived stress, emotional distress, anxiety, and depression symptoms during the early stage of the COVID-19 pandemic. This program can be a cost-effective therapeutic choice for individuals with emotional distress. Moreover, we found mindfulness may be a mechanism through which the intervention is associated with psychological health.

## Supplementary Information

**Additional file 1.** Standardized Path Coefficients of RI-CLPM between FFMQ subscales and other psychological variables.

## Data Availability

The datasets used and/or analysed during the current study are available from the corresponding author on reasonable request.

## Abbreviations

ACT: Acceptance and Commitment Therapy
CFI: Comparative fit index
COVID-19: Corona Virus disease 2019
CPSS: Chinese Perceived Stress Scale
K10: Kessler Psychological Distress Scale
OASIS: Overall Anxiety Severity and Impairment Scale
ODSIS: Overall Depression Severity and Impairment Scale
FFMQ: Short Form of Chinese Version of Five-factor Mindfulness Questionnaire
FFMQact: Act with awareness subscale of Short Form of Chinese Version of Five-factor Mindfulness Questionnaire
FFMQdes: Describe subscale of Short Form of Chinese Version of Five-factor Mindfulness Questionnaire
FFMQnj: Nonjudge subscale of Short Form of Chinese Version of Five-factor Mindfulness Questionnaire
FFMQobs: Observe subscale of Short Form of Chinese Version of Five-factor Mindfulness Questionnaire
FFMQnrt: Nonreact subscale of Short Form of Chinese Version of Five-factor Mindfulness Questionnaire
MBCT: Mindfulness-based cognitive therapy
MBI: Mindfulness-based intervention
MBSR: Minduflness-based stress reduction
MIED: Mindfulness intervention for emotional distress
RI-CLPM: Random intercepts cross-lagged panel model
RMSEA: Root mean square error of approximation
SRMR: The standardized root mean square residual
TLI: Tucker–Lewis index
UP: The Unified Protocol for the Treatment of Emotional Disorders
