# Supplementary material for: Effectiveness and mechanism of a 4-week online self-help mindfulness intervention among individuals with emotional distress during COVID-19 in China
Source: BMC Psychol. 2022 Jun 13;10:149. doi: 10.1186/s40359-022-00831-7 (PMC9190451; doi:10.1186/s40359-022-00831-7)
Supplement: Supplementary file 1 — Additional file 1. Standardized Path Coefficients of RI-CLPM between FFMQ subscales and other psychological variables. [file 40359_2022_831_MOESM1_ESM.docx]

Supplementary Data

*Table s1 Standardized Path Coefficients of RI-CLPM between Observing and Other Psychological Variables*

|  |  | Intervention Group | | | | Control Group | | | |
| --- | --- | --- | --- | --- | --- | --- | --- | --- | --- |
|  |  | W1-W2 | W2-W3 | W3-W4 | W4-W5 | W1-W2 | W2-W3 | W3-W4 | W4-W5 |
| FFMQobs-K10 |  |  |  |  |  |  |  |  |  |
|  | Stability paths |  |  |  |  |  |  |  |  |
|  | FFMQobs | 0.027 | -0.325 | 0.057 | 0.482*** | 0.091 | 0.065 | 0.187 | 0.398*** |
|  | K10 | 0.158 | 0.467** | 0.587*** | 0.689*** | 0.160 | 0.158 | 0.412*** | 0.457*** |
|  | Cross-lagged paths |  |  |  |  |  |  |  |  |
|  | FFMQobs → K10 | 0.033 | 0.145 | 0.120 | -0.160* | 0.103 | -0.106 | 0.067 | 0.003 |
|  | K10 → FFMQobs | 0.005 | -0.092 | -0.160 | -0.140 | -0.114 | 0.113 | 0.043 | -0.116 |
| FFMQobs-CPSS |  |  |  |  |  |  |  |  |  |
|  | Stability paths |  |  |  |  |  |  |  |  |
|  | FFMQobs | 0.085 | -0.018 | 0.045 | 0.475*** | 0.057 | 0.046 | 0.140 | 0.384*** |
|  | CPSS | 0.081 | -0.014 | 0.044 | 0.339* | 0.156 | 0.294** | 0.436*** | 0.407*** |
|  | Cross-lagged paths |  |  |  |  |  |  |  |  |
|  | FFMQobs → CPSS | 0.187 | -0.298 | -0.186 | -0.349** | -0.016 | 0.065 | -0.063 | -0.104 |
|  | CPSS → FFMQobs | 0.170 | 0.216 | -0.309 | -0.155 | 0.068 | 0.001 | -0.112 | 0.014 |
| FFMQobs-OASIS |  |  |  |  |  |  |  |  |  |
|  | Stability paths |  |  |  |  |  |  |  |  |
|  | FFMQobs | 0.098 | -0.088 | 0.024 | 0.550*** | -0.002 | 0.044 | 0.222 | 0.417*** |
|  | OASIS | 0.367 | 0.270 | 0.530** | 0.410** | 0.286* | 0.352** | 0.473 | 0.438*** |
|  | Cross-lagged paths |  |  |  |  |  |  |  |  |
|  | FFMQobs → OASIS | 0.170 | -0.404* | 0.035 | -0.223* | 0.029 | -0.168 | -0.028 | 0.102 |
|  | OASIS → FFMQobs | 0.067 | -0.002 | -0.412 | -0.110 | 0.187 | 0.045 | 0.010 | -0.016 |
| FFMQobs-ODSIS |  |  |  |  |  |  |  |  |  |
|  | Stability paths |  |  |  |  |  |  |  |  |
|  | FFMQobs | 0.082 | 0.097 | 0.036 | 0.529*** | 0.051 | 0.016 | 0.185 | 0.405*** |
|  | ODSIS | 0.370** | 0.317* | 0.289 | 0.174 | 0.303** | 0.175 | 0.240 | 0.224* |
|  | Cross-lagged paths |  |  |  |  |  |  |  |  |
|  | FFMQobs → ODSIS | 0.055 | -0.396* | -0.155 | -0.300* | -0.023 | 0.079 | 0.014 | 0.291** |
|  | ODSIS → FFMQobs | -0.013 | -0.126 | -0.567** | -0.171 | 0.076 | 0.183 | 0.175 | 0.053 |

*Table s2 Standardized Path Coefficients of RI-CLPM between Describing and Other Psychological Variables*

|  |  | Intervention Group | | | | Control Group | | | |
| --- | --- | --- | --- | --- | --- | --- | --- | --- | --- |
|  |  | W1-W2 | W2-W3 | W3-W4 | W4-W5 | W1-W2 | W2-W3 | W3-W4 | W4-W5 |
| FFMQdes-K10 |  |  |  |  |  |  |  |  |  |
|  | Stability paths |  |  |  |  |  |  |  |  |
|  | FFMQdes | -0.536 | -0.036 | 0.506*** | 0.599*** | -0.213 | 0.092 | 0.374*** | 0.484*** |
|  | K10 | 0.243 | 0.514*** | 0.400** | 0.670*** | 0.154 | 0.146 | 0.372*** | 0.453*** |
|  | Cross-lagged paths |  |  |  |  |  |  |  |  |
|  | FFMQdes → K10 | -0.205 | 0.056 | -0.360** | -0.121 | 0.076 | -0.286* | -0.110 | -0.047 |
|  | K10 → FFMQdes | 0.429 | -0.392* | -0.100 | -0.198* | -0.026 | -0.079 | -0.085 | -0.133* |
| FFMQdes-CPSS |  |  |  |  |  |  |  |  |  |
|  | Stability paths |  |  |  |  |  |  |  |  |
|  | FFMQdes | -0.787 | -0.061 | 0.422** | 0.635*** | -0.160 | 0.066 | 0.384*** | 0.527*** |
|  | CPSS | 0.051 | -0.018 | -0.190 | 0.360** | 0.154 | 0.296** | 0.412*** | 0.368*** |
|  | Cross-lagged paths |  |  |  |  |  |  |  |  |
|  | FFMQdes → CPSS | 0.421 | -0.035 | -0.679*** | -0.411*** | -0.144 | 0.074 | -0.050 | -0.100 |
|  | CPSS → FFMQdes | 0.495 | -0.142 | -0.231 | -0.119 | -0.005 | -0.192 | -0.108 | 0.059 |
| FFMQdes-OASIS |  |  |  |  |  |  |  |  |  |
|  | Stability paths |  |  |  |  |  |  |  |  |
|  | FFMQdes | -0.743 | -0.092 | 0.500*** | 0.631*** | -0.230 | 0.104 | 0.402*** | 0.504*** |
|  | OASIS | 0.344** | 0.282* | 0.354* | 0.398** | 0.277* | 0.335** | 0.492*** | 0.443*** |
|  | Cross-lagged paths |  |  |  |  |  |  |  |  |
|  | FFMQdes → OASIS | 0.048 | -0.018 | -0.320* | -0.247* | 0.011 | -0.169 | -0.081 | 0.016 |
|  | OASIS → FFMQdes | 0.355 | -0.156 | -0.138 | -0.173* | 0.239 | 0.109 | -0.092 | -0.032 |
| FFMQdes-ODSIS |  |  |  |  |  |  |  |  |  |
|  | Stability paths |  |  |  |  |  |  |  |  |
|  | FFMQdes | -0.567 | -0.091 | 0.486*** | 0.623*** | -0.142 | 0.083 | 0.359*** | 0.489*** |
|  | ODSIS | 0.404*** | 0.429** | 0.323 | 0.180 | 0.300** | 0.132 | 0.154 | 0.186 |
|  | Cross-lagged paths |  |  |  |  |  |  |  |  |
|  | FFMQdes → ODSIS | -0.071 | -0.039 | -0.200 | -0.321* | -0.197 | -0.063 | 0.072 | 0.043 |
|  | ODSIS → FFMQdes | 0.001 | -0.258 | -0.243* | -0.191* | 0.092 | -0.024 | 0.014 | -0.033 |

*Table s3 Standardized Path Coefficients of RI-CLPM between Act with Awareness and Other Psychological Variables*

|  |  | Intervention Group | | | | Control Group | | | |
| --- | --- | --- | --- | --- | --- | --- | --- | --- | --- |
|  |  | W1-W2 | W2-W3 | W3-W4 | W4-W5 | W1-W2 | W2-W3 | W3-W4 | W4-W5 |
| FFMQact-K10 |  |  |  |  |  |  |  |  |  |
|  | Stability paths |  |  |  |  |  |  |  |  |
|  | FFMQact | 0.076 | -0.310 | 0.278 | 0.446*** | 0.187 | -0.073 | 0.046 | 0.213* |
|  | K10 | 0.189 | 0.526*** | 0.507*** | 0.707*** | 0.055 | 0.145 | 0.397*** | 0.465*** |
|  | Cross-lagged paths |  |  |  |  |  |  |  |  |
|  | FFMQact → K10 | -0.120 | 0.001 | -0.116 | -0.053 | -0.139 | -0.057 | -0.090 | -0.041 |
|  | K10 → FFMQact | -0.008 | -0.520** | -0.108 | -0.051 | 0.025 | 0.040 | -0.278 | -0.060 |
| FFMQact-CPSS |  |  |  |  |  |  |  |  |  |
|  | Stability paths |  |  |  |  |  |  |  |  |
|  | FFMQact | -0.006 | -0.121 | 0.329* | 0.513*** | 0.206 | -0.091 | -0.099 | 0.141 |
|  | CPSS | -0.012 | 0.009 | 0.153 | 0.456*** | 0.144 | 0.325** | 0.467*** | 0.397*** |
|  | Cross-lagged paths |  |  |  |  |  |  |  |  |
|  | FFMQact → CPSS | -0.051 | -0.178 | -0.238 | -0.321*** | -0.043 | -0.008 | -0.028 | -0.107 |
|  | CPSS → FFMQact | -0.057 | -0.130 | -0.060 | 0.045 | 0.228 | -0.245* | -0.515*** | -0.235* |
| FFMQact-OASIS |  |  |  |  |  |  |  |  |  |
|  | Stability paths |  |  |  |  |  |  |  |  |
|  | FFMQact | 0.047 | -0.273 | 0.266 | 0.491*** | 0.254* | -0.066 | 0.028 | 0.160 |
|  | OASIS | 0.352** | 0.215 | 0.354* | 0.380** | 0.096 | 0.341** | 0.526*** | 0.496*** |
|  | Cross-lagged paths |  |  |  |  |  |  |  |  |
|  | FFMQact → OASIS | 0.063 | 0.090 | -0.178 | -0.238* | -0.065 | 0.027 | 0.079 | -0.004 |
|  | OASIS → FFMQact | 0.077 | -0.052 | -0.066 | 0.143 | 0.138 | 0.092 | -0.128 | -0.101 |
| FFMQact-ODSIS |  |  |  |  |  |  |  |  |  |
|  | Stability paths |  |  |  |  |  |  |  |  |
|  | FFMQact | 0.026 | -0.354 | 0.275 | 0.509*** | 0.212 | -0.034 | 0.044 | 0.172 |
|  | ODSIS | 0.370*** | 0.422** | 0.235 | 0.161 | 0.190 | 0.125 | 0.278 | 0.290* |
|  | Cross-lagged paths |  |  |  |  |  |  |  |  |
|  | FFMQact → ODSIS | -0.054 | 0.043 | -0.176 | -0.289* | -0.072 | 0.057 | 0.188 | -0.094 |
|  | ODSIS → FFMQact | -0.114 | -0.286 | -0.047 | 0.216 | -0.013 | 0.043 | -0.051 | -0.059 |

*Table s4 Standardized Path Coefficients of RI-CLPM between Non-judgment and Other Psychological Variables*

|  |  | Intervention Group | | | | Control Group | | | |
| --- | --- | --- | --- | --- | --- | --- | --- | --- | --- |
|  |  | W1-W2 | W2-W3 | W3-W4 | W4-W5 | W1-W2 | W2-W3 | W3-W4 | W4-W5 |
| FFMQnj-K10 |  |  |  |  |  |  |  |  |  |
|  | Stability paths |  |  |  |  |  |  |  |  |
|  | FFMQnj | -0.009 | 0.251 | 0.491*** | 0.729*** | 0.044 | 0.258* | 0.434*** | 0.383*** |
|  | K10 | 0.171 | 0.440** | 0.662*** | 0.763*** | 0.182 | 0.126 | 0.379*** | 0.481*** |
|  | Cross-lagged paths |  |  |  |  |  |  |  |  |
|  | FFMQnj → K10 | -0.260 | -0.233 | 0.169 | 0.048 | 0.050 | -0.093 | -0.102 | 0.082 |
|  | K10 → FFMQnj | -0.192 | -0.096 | -0.220 | 0.025 | -0.211 | -0.018 | -0.021 | 0.076 |
| FFMQnj-CPSS |  |  |  |  |  |  |  |  |  |
|  | Stability paths |  |  |  |  |  |  |  |  |
|  | FFMQnj | -0.184 | 0.222 | 0.510*** | 0.684*** | 0.024 | 0.274* | 0.47*** | 0.369*** |
|  | CPSS | 0.038 | 0.075 | 0.342* | 0.565*** | 0.283** | 0.241* | 0.434*** | 0.365 |
|  | Cross-lagged paths |  |  |  |  |  |  |  |  |
|  | FFMQnj → CPSS | -0.083 | -0.120 | 0.232 | -0.033 | 0.126 | -0.271* | 0.038 | 0.050 |
|  | CPSS → FFMQnj | -0.281 | 0.163 | -0.042 | -0.021 | -0.280* | -0.073 | 0.068 | -0.060 |
| FFMQnj-OASIS |  |  |  |  |  |  |  |  |  |
|  | Stability paths |  |  |  |  |  |  |  |  |
|  | FFMQnj | -0.044 | 0.277* | 0.509*** | 0.723*** | 0.021 | 0.344*** | 0.468*** | 0.398*** |
|  | OASIS | 0.356** | 0.281 | 0.575*** | 0.506*** | 0.376** | 0.384*** | 0.497*** | 0.456*** |
|  | Cross-lagged paths |  |  |  |  |  |  |  |  |
|  | FFMQnj → OASIS | -0.060 | -0.149 | 0.134 | -0.015 | 0.197 | 0.104 | 0.055 | 0.149* |
|  | OASIS → FFMQnj | -0.196 | -0.014 | -0.152 | 0.043 | -0.337* | 0.112 | 0.185* | 0.098 |
| FFMQnj-ODSIS |  |  |  |  |  |  |  |  |  |
|  | Stability paths |  |  |  |  |  |  |  |  |
|  | FFMQnj | -0.058 | 0.304* | 0.543*** | 0.670*** | -0.011 | 0.298** | 0.424*** | 0.354*** |
|  | ODSIS | 0.440*** | 0.402** | 0.380* | 0.270 | 0.326** | 0.092 | 0.178 | 0.179 |
|  | Cross-lagged paths |  |  |  |  |  |  |  |  |
|  | FFMQnj → ODSIS | 0.088 | -0.153 | -0.043 | 0.036 | -0.002 | -0.123 | -0.068 | 0.167 |
|  | ODSIS → FFMQnj | -0.163 | 0.079 | -0.051 | -0.124 | -0.352** | 0.039 | 0.085 | 0.029 |

*Table s5 Standardized Path Coefficients of RI-CLPM between Non-reactivity and Other Psychological Variables*

|  |  | Intervention Group | | | | Control Group | | | |
| --- | --- | --- | --- | --- | --- | --- | --- | --- | --- |
|  |  | W1-W2 | W2-W3 | W3-W4 | W4-W5 | W1-W2 | W2-W3 | W3-W4 | W4-W5 |
| FFMQnrt-K10 |  |  |  |  |  |  |  |  |  |
|  | Stability paths |  |  |  |  |  |  |  |  |
|  | FFMQnrt | 0.072 | 0.281* | 0.304** | 0.311** | -0.127 | -0.147 | 0.281** | 0.204* |
|  | K10 | 0.255 | 0.534*** | 0.563*** | 0.739*** | 0.212 | 0.236 | 0.431*** | 0.467*** |
|  | Cross-lagged paths |  |  |  |  |  |  |  |  |
|  | FFMQnrt→ K10 | -0.002 | -0.016 | -0.154 | 0.016 | -0.087 | 0.095 | -0.070 | -0.217** |
|  | K10 → FFMQnrt | -0.143 | -0.072 | 0.053 | -0.401*** | 0.318* | -0.185 | -0.149 | -0.244** |
| FFMQnrt-CPSS |  |  |  |  |  |  |  |  |  |
|  | Stability paths |  |  |  |  |  |  |  |  |
|  | FFMQnrt | 0.108 | 0.335** | 0.271* | 0.222* | -0.012 | -0.184 | 0.258** | 0.189* |
|  | CPSS | 0.064 | 0.096 | 0.200 | 0.437 | 0.162 | 0.359** | 0.500*** | 0.444*** |
|  | Cross-lagged paths |  |  |  |  |  |  |  |  |
|  | FFMQnrt → CPSS | -0.102 | -0.217 | -0.271* | -0.291** | -0.003 | 0.123 | 0.004 | -0.053 |
|  | CPSS → FFMQnrt | -0.032 | -0.149 | -0.178 | -0.497 | 0.295* | -0.180 | -0.113 | -0.041 |
| FFMQnrt-OASIS |  |  |  |  |  |  |  |  |  |
|  | Stability paths |  |  |  |  |  |  |  |  |
|  | FFMQnrt | 0.048 | 0.283 | 0.269* | 0.359*** | -0.122 | -0.111 | 0.332*** | 0.222** |
|  | OASIS | 0.368*** | 0.311* | 0.542*** | 0.456*** | 0.255 | 0.349** | 0.535*** | 0.459*** |
|  | Cross-lagged paths |  |  |  |  |  |  |  |  |
|  | FFMQnrt → OASIS | 0.069 | -0.117 | -0.011 | -0.166 | 0.012 | -0.024 | 0.070 | -0.001 |
|  | OASIS → FFMQnrt | -0.117 | 0.055 | -0.155 | -0.270* | 0.315* | -0.201 | -0.004 | -0.097 |
| FFMQnrt -ODSIS |  |  |  |  |  |  |  |  |  |
|  | Stability paths |  |  |  |  |  |  |  |  |
|  | FFMQnrt | 0.048 | 0.266 | 0.258* | 0.322** | -0.009 | -0.132 | 0.338*** | 0.216** |
|  | ODSIS | 0.397*** | 0.426*** | 0.432** | 0.207 | 0.291** | 0.130 | 0.247 | 0.213 |
|  | Cross-lagged paths |  |  |  |  |  |  |  |  |
|  | FFMQnrt → ODSIS | 0.001 | -0.142 | -0.015 | -0.228 | -0.126 | 0.271** | 0.037 | 0.124 |
|  | ODSIS → FFMQnrt | -0.151 | -0.042 | -0.202 | -0.305** | 0.163 | -0.116 | 0.099 | 0.032 |
